# Directional enhancement of photoluminescence from phosphor plates with TiO_2_ nanoantenna stickers

**DOI:** 10.1515/nanoph-2025-0419

**Published:** 2025-12-01

**Authors:** Hongjie Gao, Joshua T.Y. Tse, Shunsuke Murai, Katsuhisa Tanaka

**Affiliations:** Kyoto University, Kyoto, Japan; 12936 Osaka Metropolitan University, Sakai, Japan

**Keywords:** metasurface, sticker, photoluminescence, phosphor, distributed Bragg reflector

## Abstract

Directional illumination is critical for next-generation solid-state lighting. In this study, we demonstrate that flexible nanoantenna stickers enhance photoluminescence (PL) directionality. By integrating YAG:Ce phosphor plates and distributed Bragg reflectors (DBRs), these stickers achieve controlled radiation distribution, advancing the development of compact, high-performance illumination technologies. In addition, these stickers produce a different PL output by simply changing the thickness of the phosphor plate. The output PL intensity is doubled by placing a DBR layer on the bottom of the plate, thereby transmitting blue excitation light while reflecting yellow PL. This study provides a simple and versatile method for tuning PL from phosphor plates. This technique can serve as a fundamental tool for controlling light flow with improved efficiency.

## 1 Introduction

White light-emitting diodes (LEDs) are solid-state lighting devices that emit white light using various conversion techniques. Unlike conventional incandescent bulbs and fluorescent tubes, white LEDs are engineered by combining different wavelengths, most commonly using a blue LED coated with a yellow phosphor (typically Ce^3+^-doped yttrium aluminum garnet, YAG:Ce) [1]. This technique blends blue and yellow emissions to create the perception of white light. In next-generation high-power lighting, the necessity for directional illumination devices has become increasingly evident. In this context, white laser diodes (LDs) have been intensively studied. However, LDs are directional light sources. When combined with conventional yellow phosphors that emit omnidirectionally, this creates a mismatch in their respective spatial distributions. In other words, the output directionality and intensity of PL from the phosphors are not sufficiently balanced to the blue laser light.

Nanoantennas provide an advanced way to address this directionality-mismatch issue [2], [3], [4], [5], [6], [7], [8], [9], [10], [11]. Nanoantennas can be single particles [12], [13], [14], [15], Yagi–Uda structures [5], or assembled nanostructures that form photonic bands [16], [17]. A periodic array of nanoparticles (NPs) is a typical nanoantenna type, which supports surface lattice resonance (SLR), a high-quality factor mode accompanied by a strong electromagnetic field in the lattice plane [18], [19], [20], [21], [22], [23], [24]. It emerges from the coupling between in-plane diffraction, known as the Rayleigh anomaly (RA), and the local resonance in each NP. SLR can be coupled with PL to tune PL both spatially and spectrally [7], [25], [26], [27], [28], [29]. When SLR is combined with a practical, high-quantum-yield phosphor sample, PL from the phosphor is coupled with SLR and radiated into the forward direction to achieve a homogeneous mixing with directional blue light, resulting in a mirror- and reflector-less illumination device with directional output [30], [31].

In this study, unlike conventional nanoantennas, which are directly fabricated on phosphor plates, the nanoantennas were embedded in a poly(dimethylsiloxane) (PDMS) layer, hereafter called a nanoantenna sticker [32], [33], [34], and attached to a commercial YAG:Ce phosphor plate, yielding a nanoantenna–phosphor system. The sticker is reusable, i.e., it is peeled off from one substrate and reattached to another substrate multiple times. By leveraging this property, we investigated the dependence of PL modulation on the thickness of the YAG:Ce plate. To further enhance PL, a multilayer distributed Bragg reflector (DBR) was deposited on the backside of the plates [30]. We also demonstrated the modulation of PL outcoupling patterns by changing the shape of the sticker from hexagonal to square. The color control of the forward PL was evaluated using an integrating sphere. Compared with a phosphor plate in which the nanoantenna is directly fabricated on it and suffers from a large refractive-index mismatch between superstrate (air) and substrate (YAG:Ce), our configuration provides an NP array with a lower mismatch because of the PDMS layer. This effect on the outcoupling intensity was examined via simulations.

## 2 Experiment method

### 2.1 Fabrication of samples

#### 2.1.1 Fabrication of the hexagonal pattern sticker

**Figure 1:**
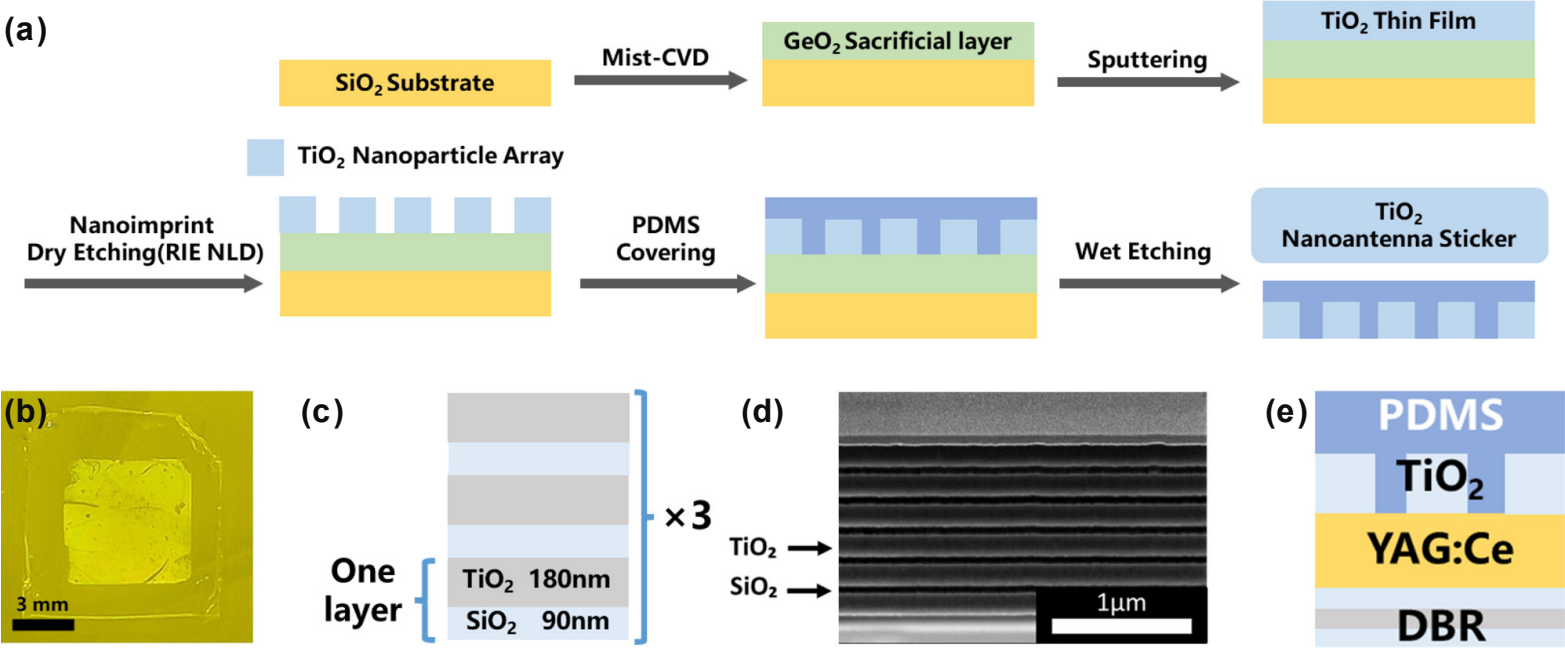
Fabrication and structural schematic of the nanoantenna stikcer–YAG:Ce–DBR system. (a) Fabrication process of the hexagonal pattern sticker (period = 420 nm, nanoantenna area = 6 × 6 mm^2^). (b) Digital photograph of the hexagonal pattern sticker attached to the YAG:Ce plate. (c) Schematic illustration of the three-layer DBR structure. (d) Cross-sectional scanning electron microscope image of the six-layer DBR. (e) Schematic illustration of the sticker–YAG:Ce–DBR system.

The hexagonal pattern TiO_2_ nanoantenna sticker was fabricated via nanoimprint lithography, followed by its transfer into the PDMS matrix. The fabrication process is shown in Figure 1a. First, a 750-nm-thick amorphous germanium dioxide (a-GeO_2_) layer was deposited onto a commercial SiO_2_ glass substrate via mist chemical vapor deposition. This a-GeO_2_ layer served as a sacrificial layer for the subsequent transfer process. To improve its water solubility and minimize oxygen vacancies (GeO_*x*_), the film was annealed in an O_2_ atmosphere at 750 °C for 10 min [35]. Then, a thin amorphous TiO_2_ film was formed via sputtering, followed by the spin-coating of a resist layer (TU7, Obducat). Then, the designed nanostructure was patterned onto the resist via nanoimprint lithography (Entre3, Obducat). The resist pattern was further transferred to the TiO_2_/a-GeO_2_ layer by combining an O_2_ ashing and neutral loop discharge etching using a CHF_3_/C_4_F_8_/Ar/O_2_ gas mixture, yielding TiO_2_ NP arrays on the a-GeO_2_ surface. For the transfer, a PDMS precursor was mixed with an appropriate curing agent, poured over the patterned surface, and cured at 80 °C for 2 h. Finally, the a-GeO_2_ sacrificial layer was dissolved in ultrapure water for 24 h, yielding a free-standing PDMS film incorporating with the hexagonal TiO_2_ nanoantenna array. A digital photo of the sticker on the YAG:Ce substrate is shown in Figure 1b. Refer to Supporting Information Figure S1 for the fabrication of square pattern stickers.

#### 2.1.2 Fabrication of DBR

The DBR was fabricated via sputtering using a multitarget sputtering system (EB1100, Canon Anelva Co., Ltd.). A 90-nm-thick SiO_2_ thin film was deposited on the backside of the YAG:Ce substrate, followed by depositing a 180-nm-thick TiO_2_ thin film. A schematic and scanning electron microscopy image of the DBR are shown in Figure 1c and d, respectively. The final sample configuration is depicted in Figure 1e.

### 2.2 Characterization

We attached the flexible nanoantenna stickers onto the YAG:Ce plates with thicknesses (*t*) ranging from 110 to 477 μm. The angle-dependent extinction and PL spectra were recorded. The quantum yield of the system and the CIE chromaticity diagram were measured using an integrating sphere. An ultraviolet–visible spectrometer was used to investigate the transmission properties of the DBR on the YAG:Ce plate.

#### 2.2.1 Optical extinction

We measured the zeroth-order extinction spectrum by illuminating the sample with a collimated beam from a halogen lamp (SLS201L/M, Thorlab). We evaluated the optical extinction (*E*), defined as *E* = 1 −*T*/*T*_0_, where *T* represents the zeroth-order transmission through the nanoantenna sticker on the phosphor plate and *T*_0_ denotes the transmission through the flat phosphor plate. The zeroth-order transmission was collected using a fiber (numerical aperture of 0.22) coupled to the spectrometer (Flame-S, Ocean Insight). The angle of incidence (*θ*_in_) was varied in the *z* − *x* plane, with the azimuthal angle from the *x*-direction being 0°.

#### 2.2.2 Photoluminescence

The nanoantenna sticker was placed on the forward side of the YAG:Ce plate, and the sample was placed on the rotation stage. A diode-pumped solid-state laser (*λ* = 440.6 nm; Shanghai Dream Lasers Technology) was incident onto the substrate side at the angle that exhibited the strongest emission in each case. The PL that radiated to the opposite side through the sticker was collected by an optical fiber coupled to the spectrometer as a function of the emission angle *θ*_em_ defined in the *z* − *x* plane. The PL enhancement was defined as the PL intensity obtained with the sticker, *I*, divided by the value obtained without the sticker, *I*_ref_, i.e., *I*/*I*_ref_.

#### 2.2.3 Integrating sphere measurements

The quantum yield and the CIE chromaticity diagram were evaluated using a calibrated integrating sphere [diameter = 10 in. (0.254 m)] (RTC-060-SF, Labsphere). An LD (*λ* = 457 nm) was incident on the sample from the substrate side. The output light was integrated throughout the sphere and collected using a fiber-coupled spectrometer. The spectral range between *λ* = 419 and 798 nm was used for the chromaticity calculation. The color coordinates were determined using the CIE 1931 XYZ chromaticity system.

#### 2.2.4 Simulation

Finite-difference time-domain (FDTD) simulations were performed using a model consisting of cylinder NPs made of TiO_2_ with a diameter of 200 nm and a height of 150 nm. The YAG:Ce substrate and the PDMS superstrate were semi-infinite layers with refractive index, *n* = 1.820 and 1.415, respectively. A 20 nm PDMS layer was inserted between the nanoantenna and the substrate to reproduce the experimental situation. Periodic boundary conditions were applied in the lateral direction to produce a hexagonal lattice with a periodicity of 420 nm, and perfectly matched layers were used along the *z*-direction. In all simulations, the incident plane wave was applied from the top (PDMS) side with a linear polarization along the *x*-direction. The simulations for the square lattice and a single NP were also done.

The DBR transmission and reflection were also simulated using the FDTD method. The DBR model comprised alternating layers of SiO_2_ (90 nm) and TiO_2_ (180 nm), with their refractive indices set to 1.460 and 2.400, respectively.

## 3 Results and discussion

### 3.1 PL enhancement

#### 3.1.1 Hexagonal pattern

**Figure 2:**
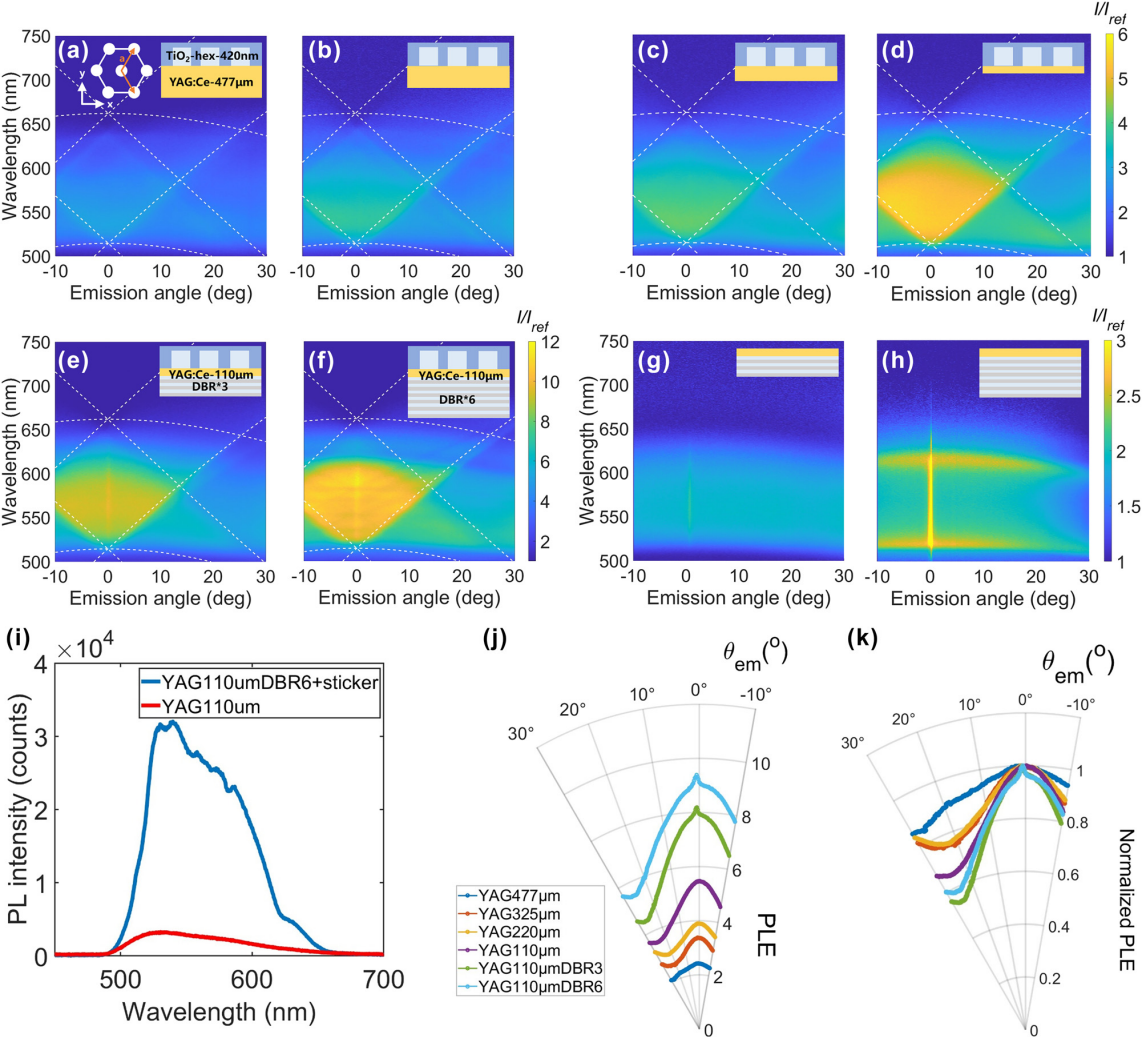
PL enhancement spectra of YAG:Ce substrates with TiO_2_ nanoantenna stickers (hexagonal pattern, period = 420 nm) detected in p-polarization. Substrate thicknesses (*t*) are (a) 477, (b) 325, (c) 220, and (d)–(h) 110 μm, the left inset in (a) shows the particle arrangement. The insets in the upper-right corners of panels (a)–(h) indicate the substrate thickness and the presence or absence of the DBR layer. (e)–(f) PL enhancement spectra for *t* = 110 μm sample with (e) three- and (f) six-layer of DBR. (g)–(h) PL enhancement spectra of the *t* = 110 μm plate with (g) three- and (h) six-layer DBR, without nanoantenna sticker. (i) PL spectra of the *t* = 110 μm plate with six-layer DBR and the hexagonal pattern sticker, in comparison to that of the bare 110 μm-thick substrates at *θ*_em_ = 0°. (j) Angular profiles of the PL enhancement spectrally integrated over *λ* = 500–650 nm. (k) Normalized angular profiles of the integrated PL enhancement.

Figure 2 summarizes the PL enhancement spectra of the YAG:Ce plate with the hexagonal pattern sticker. Here, the same sticker was placed on YAG:Ce plates with varied *t*. In each panel, the PL enhancement was plotted using the bare plate with identical *t* but without the sticker as the reference. An obvious *t* dependence was observed: as the substrate became thinner, the enhancement factor increased, accompanied by an improvement in PL directionality. The enhancement factor reached a maximum of 5.64-fold at *λ* = 548 nm for the 110-μm-thick plate. The sticker can be detached and re-attached repeatedly, and reproducible PL enhancement is obtained each time (see Supporting Information Figure S2).

The dotted lines in the panels represent the in-plane diffraction conditions or RA lines, which can be expressed as follows:

(1) $${\vec{k}}_{0}={\vec{k}}_{\text{in}}\left(\theta_{\text{in}}\right)\pm \vec{G}\left(m_{1},m_{2}\right),$$

where ${\vec{k}}_{0}$ denotes the wavevector in free space, ${\vec{k}}_{\text{in}}\left(\theta_{\text{in}}\right)$ denotes the in-plane component of the incident wavevector at an incident angle *θ*_in_, and $\vec{G}\left(m_{1},m_{2}\right)$ denotes the reciprocal lattice vector indexed by integers *m*_1_ and *m*_2_. When the incident light has no component along the *y*-direction, as in the present case, the magnitude of the free-space wavevector ${\vec{k}}_{0}$ is expressed for a hexagonal lattice as

(2) $$\begin{array}{ll} k_{0}^{2} & =k_{\text{in}}^{2}+{\left(\frac{2\pi }{a}\left(m_{1}+m_{2}\right)\right)}^{2}+\frac{1}{3}\left[{\left(\frac{2\pi }{a}\left(m_{1}+m_{2}\right)\right)}^{2}\right.\\ & \;\left.+{\left(\frac{2\pi }{a}\left(m_{1}-m_{2}\right)\right)}^{2}\right], \end{array}$$

where *a* represents the lattice constant which is 420 nm in the present sample and *m*_1_ and *m*_2_ denote the diffraction orders [26]. RA depends on *n* of the environment surrounding the nanoantenna, and RAs with *n* = 1.415 (representing PDMS) and 1.820 (YAG:Ce) are plotted in Figure 2a–f (white dotted lines). Moreover, the main enhancement was confined to the wavelength range between the two RA conditions (*n*_YAG:Ce_ and *n*_PDMS_), since there intensified outcoupling is realized [30]. While in other regions, the constraint condition induces part-depressed coupling, and thus results in lower enhancements.

These results mean that the observed PL enhancement is strongly governed by the optical scattering conditions determined by the NP arrangement. To be more precise, the confinement of the enhancement between these scattering condition wavelengths indicates that the directional output is due to coherent scattering of the PL otherwise trapped inside the plate by total internal reflectance or the mismatch of in-plane momentum (*k*_‖_). As shown in the extinction spectra in Supporting Information Figure S3, SLRs are supported in the samples even though they are not very strong. SLR is induced via coupling between the localized Mie resonance of the TiO_2_ NPs and the diffractive orders of the periodic array. In Supporting Information Figures S4 and 5, we simulated the field distributions associated with the Mie resonance of the single NP and the SLR of the periodic array, respectively. The results feature a broad Mie resonance of each NP coupling via in-plane diffraction to induce SLR. In the PL outcoupling process, SLRs couple to and redirect PL into the forward direction defined by the pattern and periodicity of the nanoantenna, thereby yielding a notable PL enhancement.

The DBR further enhances forward radiation, as evident by the comparison between Figure 2d–f. Compared with the 110-μm-thick YAG without DBR, where the maximum enhancement is 5.64-fold (Figure 2d), the introduction of three or six layers of DBRs leads to the enhancement up to 9.67- or 11.47-fold, respectively. Because DBRs are designed to transmit blue light and reflect yellow light (see Supporting Information Figures S3b, c and S6 for the spectrum), the DBRs do not reduce the excitation power of blue LDs, and the PL radiated into the backside is reflected into the forward direction. This effect of DBR is better examined in Figure 2g and h, where the PL enhancements from the plates with DBRs are plotted with a bare substrate as a reference. The enhancement is broadband in the 520–600-nm range and dispersionless, consistent with the transmission spectrum in Supporting Information. The enhancements are approximately 1.5- and 2-fold for the three- and six-layer DBRs, respectively. Given the effect of DBR, which recycles the PL radiated to the backside, the maximum value of 2-fold is reasonable. In addition, the enhancement is discontinuously high at *θ*_em_ = 0°, because the YAG:Ce plate with DBR acts as a Fabry–Perot cavity and the confinement of light is strongest at *θ*_em_ = 0°. This discontinuous enhancement is also evident after placing the sticker (Figure 2e and f).

To closely examine the change in the spectral features, Figure 2i plots the PL spectra at *θ*_em_ = 0° for the YAG:Ce plate (*t* = 110 μm) with the six-layer DBR and nanoantenna sticker, and the bare YAG:Ce plate (*t* = 110 μm). The reference shows a broadband yellow PL centered at *λ* = 540 nm, which is typical for YAG:Ce phosphor. In contrast, the PL spectrum from the antenna and DBR plate show significantly higher intensity, with several bumps arising from the spectral variation of the reflectance from the DBR. An evident bump at *λ* = 625 nm is the edge of the DBR reflectance band, and the reflectance is significantly at longer wavelengths.

The directionality of the PL enhancement is better visualized by the polar plot. In Figure 2j and k, we integrated the PL intensity between *λ* = 500 and 650 nm and then divided the integrated intensity by that for the bare substrate, to obtain the angular enhancement profiles. Figure 2j shows that the thinner substrate results in stronger enhancement with narrower angular distribution, which is further increased by the DBRs. The increased directionality becomes more evident by normalization (Figure 2k). PL output is a sum of directional components and a diffuse background. Given that the directional output is due to coherent scattering of the PL otherwise trapped inside the plate by total internal reflectance, the thinner plate contains more trapped PL photons in the detection spot. An analysis of the thickness dependence of PL directionality is found in Supporting Information Figure S7.

#### 3.1.2 Square pattern

**Figure 3:**
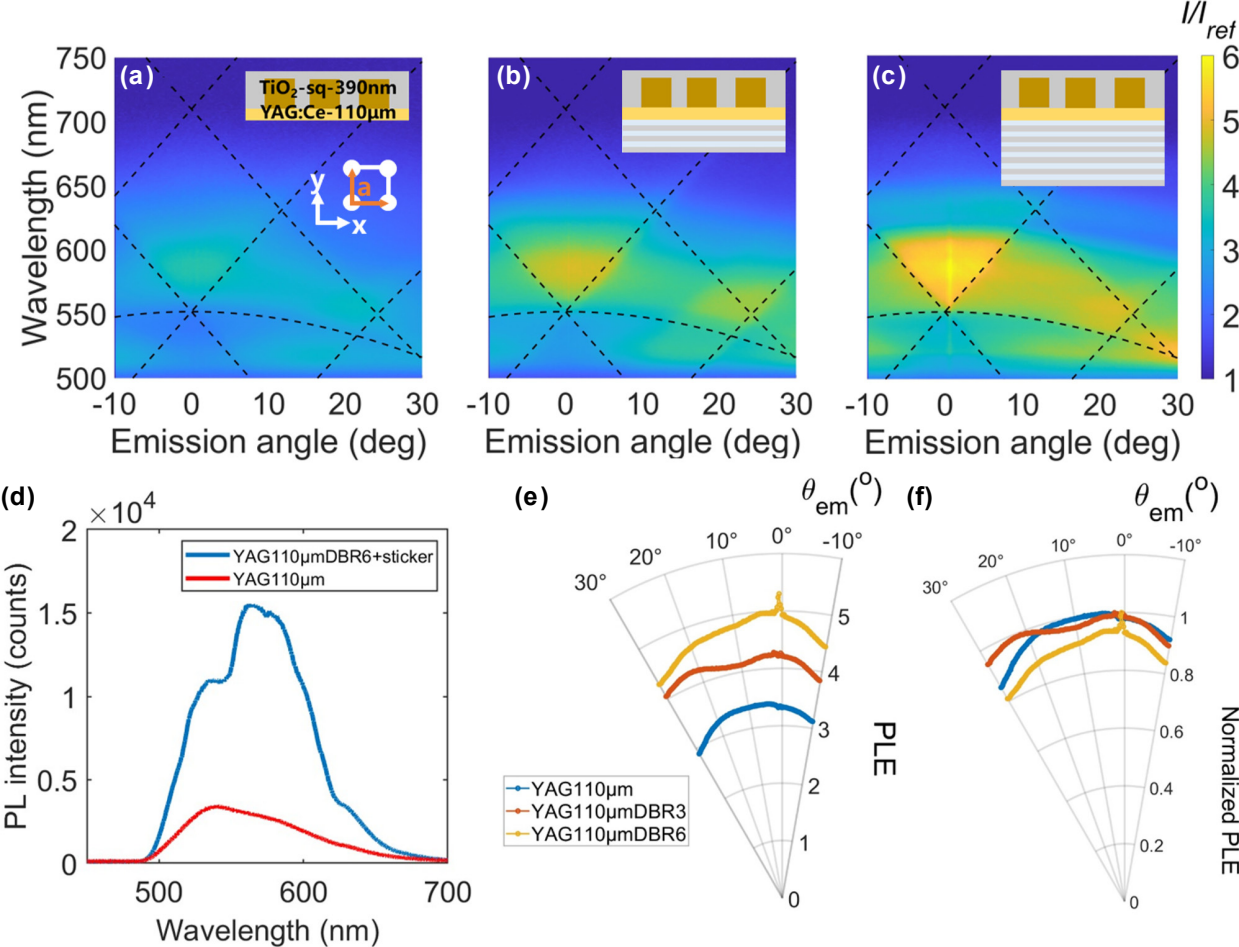
PL enhancement spectra of 110 μm-thick YAG:Ce substrates with square pattern TiO_2_ nanoantenna stickers (period = 390 nm) detected in p-polarization. DBR layer number are (a) 0, (b) 3, and (c) 6. The insets in the upper-right corners of panels (a)–(h) indicate the substrate thickness and the presence or absence of the DBR layer. (d) PL spectra of the *t* = 110 μm-plate with 6-layer DBR and the square pattern sticker, in comparison to that of the bare 110 μm-thick substrates at *θ*_em_ = 0°. (e) Angular profiles of the PL enhancement spectrally integrated over *λ* = 520–620 nm. (f) Normalized angular profiles of the integrated PL enhancement.

Subsequently, we investigated the PL enhancement using the square pattern nanoantenna sticker on the *t* = 110-μm YAG:Ce plate (see Supporting Information for fabrication of the square lattice sticker). In this case, the enhancement increases with the number of DBR layers, as shown in Figure 3a–c. The dotted lines represent the RA lines for the square lattices with *a* = 390 nm and *n* = 1.415 and 1.820. For the square lattice, the reciprocal lattice vector is given by

(3) $$k_{0}^{2}=k_{\text{in}}^{2}+{\left(\frac{2\pi }{a}m_{1}\right)}^{2}+{\left(\frac{2\pi }{a}m_{2}\right)}^{2}.$$

Compared with the hexagonal lattice, the enhancement zone shifts due to differences in both the lattice arrangement and period. Especially, the line associated with (*m*_1_, *m*_2_) = (0, ±1) diffraction order lies around 550 nm at normal incidence and shifts to 520 nm at 30°. A notable PL enhancement appears along this RA line in the whole angle range of measurements because the line is inside the spectral region of the maximum PL intensity of YAG:Ce. The effect of (0, ±1) order is evident in the spectrum at *θ*_em_ = 0° (Figure 3d), where a dip is observed around *λ* = 550 nm. The effect is also reflected on the less directional PL profile for the square pattern case (Figure 3e and f). Also the magnitude of PL enhancement is less than that for the hexagonal lattice case. This is because the square lattice is not the close packed lattice and there are less NPs in a unit cell to gather and redirect the emitted PL. We further confirm our analysis using simulation (see Supporting Information Figure S8).

The comparison of the results for hexagonal and square pattern stickers confirms that the enhancement profiles, both spatially and spectrally, can be flexibly tuned by adjusting the lattice symmetry and periodicity of the sticker.

### 3.2 Integrating sphere measurements

**Figure 4:**
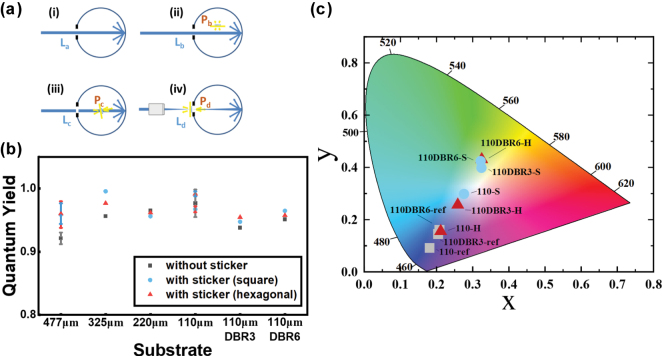
Quantum yield and CIE chromaticity measured with an integrating sphere. (a) Sketches for the measurement using an integrating sphere: (i) no sample, (ii) sample placed inside the sphere without laser direct incidence, (iii) sample placed inside the sphere with laser incident at a 10° angle, and (iv) sample positioned outside the sphere, attached to the window. (b) Quantum yield diagram for the YAG:Ce plates with varying *t*, with/without DBRs, and with/without hexagonal/square lattice stickers. The error bars are attached to the *t* = 477 and 110 μm groups. (c) CIE chromaticity diagrams of the forward output component (*L*_d_ + *P*_d_) for the plates measured in (b).

We examined the quantum yield of the plate with the sticker and DBR using an integrating sphere. The sample was positioned at different locations of the integrating sphere: Figure 4a shows the spectra of the sphere without the plate (i), with the plate and the laser beam first hitting the sphere wall (ii), with the plate and the laser beam directly hitting the plate (iii), and with the plate on the front port (iv). From the measured spectra, we derived the following quantities: the laser beam intensity (*L*_a_, *L*_b_, *L*_c_, and *L*_d_ for (i), (ii), (iii), and (iv), respectively, where the spectral range of integration is 454.6–458.8 nm) and the total PL intensity integrated over the corresponding emission wavelengths (*P*_b_, *P*_c_, and *P*_d_ for (ii), (iii), and (iv), respectively, 479–760 nm). When a fraction *μ* of the laser beam scattered from the sphere wall is absorbed by the plate, *L*_b_ and *L*_c_ are given by [36]

(4) $$L_{\text{b}}=\left(1-\mu \right)L_{\text{a}},$$

and

(5) $$L_{\text{c}}=\left(1-A\right)\left(1-\mu \right)L_{\text{a}},$$

where *A* represents the absorptance by the plate at the excitation wavelength. This absorptance can be written from Eqs. (4) and (5) as follows:

(6) $$A=1-\frac{L_{\text{c}}}{L_{\text{b}}}.$$

The total PL is given by the absorptance of the laser beam multiplied by the conversion efficiency *η*_c_ and can be expressed as follows:

(7) $$\eta_{\text{c}}L_{\text{a}}A=P_{\text{c}}-\left(1-A\right)P_{\text{b}};$$

thus,

(8) $$\eta_{\text{c}}=\frac{P_{\text{c}}-\left(1-A\right)P_{\text{b}}}{L_{\text{a}}A}.$$

The results demonstrate that all samples exhibit *η*_c_ above 0.9, regardless of whether a sticker/a DBR is presented (Figure 4b), demonstrating the effectiveness of this system for light conversion. There is a small difference between the quantum yields of the with- and without-sticker samples for a 477 μm plate. One possibility is the effect of re-absorption: Because of the high refractive index of the plate, part of the PL is trapped inside the plate by total internal reflection, and reabsorbed by the plate before escaping from the side. The sticker extracts the PL before being re-absorbed, which could contribute to increasing the quantum yield. This re-absorption argument aligns with the experimental observation that the thicker bare plate shows the lower quantum yield. Even with a small deviation, the quantum yield remains high and almost unchanged, meaning that the total amount of PL radiated does not increase so much by the presence of sticker. This is the same for the plates with directly fabricated nanoantenna, wherein the nanoantenna modifies the spatial distribution of PL via directional outcoupling [37].

Based on the measurements obtained using the sample positioned outside the window of the sphere (*P*_d_), the CIE chromaticity diagram is plotted in Figure 4c. These data correspond to the YAG:Ce plate with *t* = 110 μm. For the bare plate, the output is represented by the blue region with the coordinate (*x*, *y*) = (0.18, 0.09). With the DBR, the chromaticity shifts toward the white region but remains in the blue region (*x*, *y*) = (0.21, 0.16) for the six-layer DBR. In contrast, both the square and hexagonal pattern stickers enhance the conversion of blue light to yellow light, and the coordinate shifts toward the white region. In addition, increasing the number of DBR layers shifts the emission further toward yellow; however, excessive layering pushes the chromaticity away from the white-light region, resulting in an overly yellow emission. The coordinates for the six-layer DBR plate with hexagonal and square lattice stickers are (*x*, *y*) = (0.33, 0.43) and (*x*, *y*) = (0.32, 0.42), respectively.

### 3.3 Numerical simulation of PL enhancement

**Figure 5:**
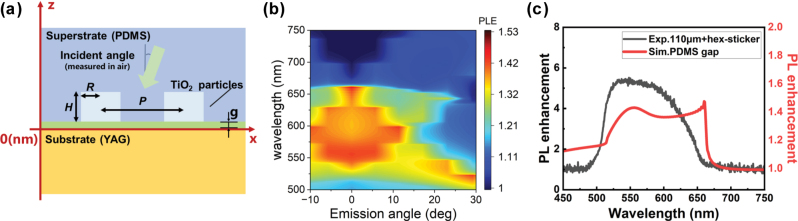
Simulation analysis of PL enhancement of YAG:Ce–nanoantenna sticker system. (a) Schematic of the simulation structure of the TiO_2_ nanoantenna sticker with a 20 nm PDMS gap between the sticker and the YAG:Ce substrate. (b) Simulated PL enhancement spectrum of the hexagonal pattern sticker on the YAG:Ce substrate. (c) Comparison of the experimental PL enhancement using a 110 μm-thick substrate with a hexagonal pattern sticker (gray line) and the simulated result (red line) at 0° incidence.

We simulated the PL enhancement by using reciprocal relation between the field enhancement inside the active volume and PL. In the present system, the active layer is the YAG:Ce plate, i.e., the PL is generated inside the excited volume in the phosphor plate. Thus, the active volume extends from the bottom to the top of the plate. Thus, in order to simulate the PL enhancement by reciprocity, *z* needs to be extended to the whole volume of the plate. In order to save computational costs, we limited the calculation region to –500 nm ≤ *z* ≤ 0 nm (the active region closest to the antenna) and accumulated the squared electric field over the unit cell (see Figure 5a for the simulation model).

The calculated PL enhancement (Figure 5b) follows the RA conditions as in the case for experiments in Figure 2. It is noted that the simulated electric field is normalized to the incident field and thus the simulated magnitudes do not quantitatively agree with the experimental values.

We further compared the simulation data with the experimental result at *θ*_em_ = 0° in Figure 5c. The enhancement is notable between *λ* = 500 and 650 nm, corresponding to RA conditions with *n* = 1.415 (PDMS) and 1.82 (TiO_2_). The spectral shape of the simulated profile is in global agreement with the experiment. Experimental PL enhancement does not show local maximum at the longer edge of RA around *λ* = 650 nm, because of the insufficient scattering at this wavelength. This is probably due to the imperfect shape of TiO_2_ from the cylinder and/or unintended gap between the phosphor plate and the sticker.

We also examined the effect of PDMS layer on PL enhancement numerically (see Supporting Informa tion Figure S9). Compared to the air superstrate, the PDMS superstrate gives a higher PL enhancement at around *λ* = 650 nm, an RA associated to *n* of YAG:Ce.

## 4 Conclusions

In this study, we characterized the optical properties of TiO_2_ nanoantenna stickers on YAG:Ce plates combined with multilayer DBRs and demonstrated tailored PL both spatially and spectrally. We found that PL enhancement strongly depends on the substrate thickness and the number of DBR layers. Using hexagonal pattern stickers, the enhancement factor increases as the substrate becomes thinner, reaching a maximum of over 11-fold with a 110-μm-thick substrate and a six-layer DBR. The PL directionality is also improved under these conditions. Furthermore, the integrating sphere measurements indicate that all samples maintain high quantum yields (>0.9) . Both square and hexagonal pattern stickers yellow-shift the output color toward the white region, whereas excessive DBR layering can push the chromaticity further into the yellow region. Overall, our results demonstrate that the combination of patterned nanoantenna stickers and DBRs provides a versatile platform for controlling the PL intensity, spectral distribution, and directionality in phosphor-based light-emitting systems. This approach offers a promising strategy for achieving highly efficient and spectrally tunable light conversion in compact devices.

## Supplementary Material

Supplementary Material Details
