# Supplementary material for: Directional enhancement of photoluminescence from phosphor plates with TiO2 nanoantenna stickers
Source: Nanophotonics. 2025 Dec 1;14(25):4627–36. doi: 10.1515/nanoph-2025-0419 (PMC12714061; doi:10.1515/nanoph-2025-0419)
Supplement: Supplementary file 1 — Supplementary Material Details [file j_nanoph-2025-0419_suppl_001.pdf]

# Supporting Information: Directional Enhancement of Photoluminescence from Phosphor Plates With $TiO_2$ Nanoantenna Stickers

Hongjie Gao,[†] Joshua T. Y. Tse,[‡] Shunsuke Murai,*[‡] and Katsuhisa Tanaka[†]

†*Department of Material Chemistry, Graduate School of Engineering, Kyoto University, Katsura, Nishikyo 6158510, Japan*

‡*Department of Physics and Electronics, Graduate School of Engineering, Osaka Metropolitan University, Osaka, 5998531, Japan*

E-mail: murai@omu.ac.jp

Phone: +81 (0)72 2476210

## Contents

**1 Fabrication of square lattice nanoantenna stickers** **S3**

**2 Variation in PL intensity with several detach/re-attach cycles** **S4**

**3 Extinction spectra** **S5**

**4 Simulated field distribution for the single nanoparticle** **S7**

**5 Simulated field distribution for the nanoparticle array** **S9**

**6 Comparison of DBR spectra between experiment and simulation S10**

**7 Analysis of thickness dependence of PL directionality S11**

**8 Simulated PL enhancement of the square pattern nanoantenna sticker S12**

**9 Influence of PDMS layer on PL Enhancement S13**

# 1 Fabrication of square lattice nanoantenna stickers

The $TiO_2$ nanoantenna sticker was fabricated by nanoimprint lithography followed by the transfer of the nanoantenna into the PDMS matrix. The fabrication process is schematically shown in Fig. S1a. The process is similar to that for the hexagonal sticker, but with different deposition techniques of amorphous germanium dioxide (a-$GeO_2$) and $TiO_2$.

Firstly, a 750 nm-thick a-$GeO_2$ layer was deposited onto a commercial $SiO_2$ glass substrate via electron-beam evaporation. To improve its water solubility and minimize oxygen vacancies ($GeO_x$), the film was annealed in an $O_2$ atmosphere at 750°C for 10 min. Subsequently, a 150 nm-thick titanium (Ti) film was deposited using electron-beam evaporation. A resist layer (TU7, Obducat) was then spin-coated, and the resist was nanopatterned by nanoimprint lithography (Entre3, Obducat). The unmasked Ti/a-$GeO_2$ regions were etched via reactive ion etching with an Ar/$Cl_2$ gas mixture, forming Ti nanoparticle arrays on the a-$GeO_2$ surface. These Ti nanoparticles were converted into $TiO_2$ nanodisks by thermal oxidation at 750°C for 10 min. The fabricated sticker has a 6 mm × 6 mm area (see Fig. S1b), the same sa that of the hexagonal patterded sticker.

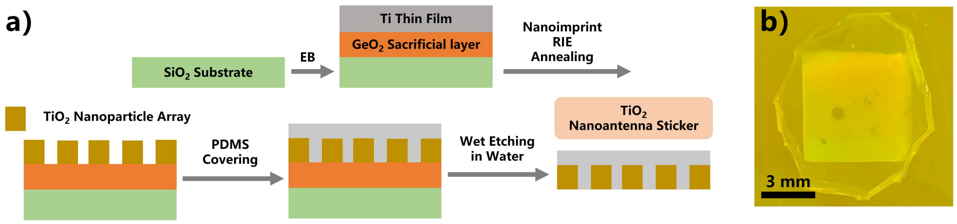


Figure S 1: (a) The fabricating process of the square pattern sticker (period = 390 nm). (b) The digital photo of the squarepattern sticker.

## 2 Variation in PL intensity with several detach/re-attach cycles

Figure S2 shows that the PL enhancement remains almost unchanged after several detach/re-attach cycles, demonstrating the reliability and effectiveness of the sticker-based nanoantenna system.

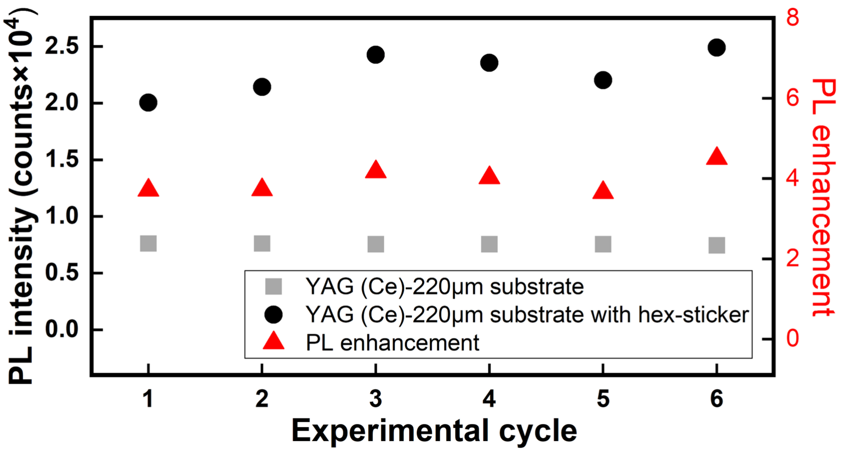


Figure S 2: Diagram of the highest PL intensity (without background subtraction) of the flat YAG:Ce plate (gray squares) and the plate with the hexagonal pattern $TiO_2$ nanoantenna sticker (black dots) at $\theta_{em}$=0° for each detach/re-attach cycle. The red triangles indicate the highest PL enhancement values for each cycle.

# 3 Extinction spectra

Figure S3 presents the optical extinction spectra of the hexagonal patterned $TiO_2$ nanoantenna sticker placed on YAG:Ce plates with zero, three, and six layers of DBRs on the backside, as well as those of YAG:Ce plates with three and six DBR layers without the sticker. The Rayleigh anomaly (RA) conditions are indicated by dashed lines in the spectra measured with the nanoantenna sticker.

The sticker on the YAG:Ce plate without DBR (Figs. S3a and d for p and s-polarization), a broad and dispersionless extinction appears in the shorter wavelengths area which is the Mie resonance localized in each $TiO_2$ nanoparticles. Note that the reference is the neat YAG:Ce plate and thus the absorption of $Ce^{3+}$ is subtracted from the extinction. In the longer wavelengths region of this broad extinction, some features follow the RA lines. This indicates the coupling between local Mie resonances via diffraction, i.e., the formation of SLRs.

In the extinction spectra of YAG:Ce plate with three- or six-layer DBR in Fig. S3b and c, a pronounced and broad extinction feature appears in the $520-600$ nm range, overlapping with the YAG:Ce emission band. This feature will be compared with simulation in Fig. S6. The dispersive features following the RA are also observed in the spectra of the sticker on the YAG:Ce with DBRs (Figs. S3e and f for three-and six-layer DBR). In Figs. S3e and f, the influence of DBRs was subtracted by using a plate with DBR as the reference. For the six-layer DBR sample, the extinction effect of the DBR is so pronounced so that the diffraction-related features are almost completely suppressed.

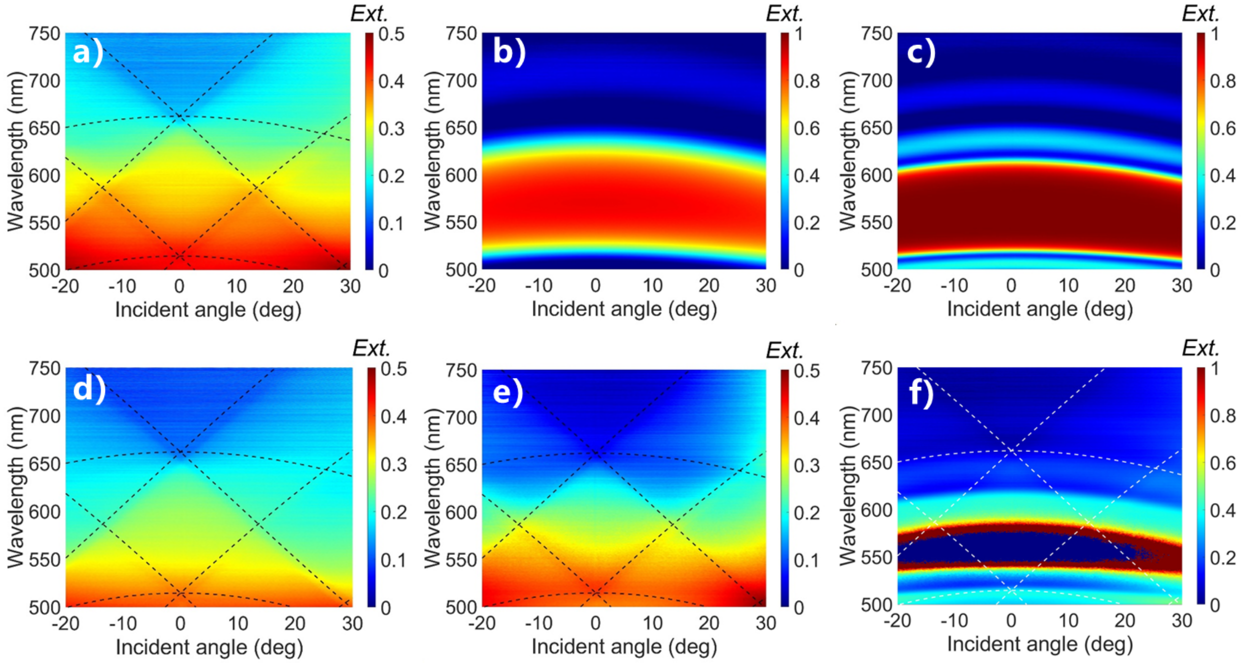


Figure S 3: Extinction spectra of 110 $\mu$m-thick YAG:Ce plates with $TiO_2$ nanoantenna stickers (hexagonal pattern, period = 420 nm) and/or DBRs. (a,d) Plate with the sticker and no DBRs under (a) p- and (d) s-polarization, with the neat plate as the reference. (b, d) The plates with (b) 3- and (c) 6-layer DBR without the sticker, with the neat plate as the reference. (e, f) Plates with the sticker and (e) 3 and (f) 6-layer DBR, with the plate with 3 and 6-layer DBR as the references.

# 4 Simulated field distribution for the single nanoparticle

The electric field distribution was simulated for a single nanoparticle, diameter 200 nm and height 150 nm of $TiO_2$ cylinder on YAG:Ce substrate. The model volume was 400 nm $\times$ 400 nm $\times$ 500 nm in x, y, and z-directions, surrounded by perfect matching layers. Figure S4a shows the electric field intensity integrated over the simulated volume. The field intensity shows a broad peak corresponding to the dipole mode, which overlaps the wavelength range between the two RA conditions ($\lambda$ at 515 and 661 nm as shown in the next section). The scattering cross section increases toward shorter wavelengths (Fig. S4b), accounting for the relatively strong extinction at $\lambda$ below 600 nm (Fig. S3a). The electric field intensity distributions on the $x - y$ (upper panel) and $y - z$ (lower panel) planes are shown in Fig. S4c, exhibiting characteristic of dipole Mie resonance.

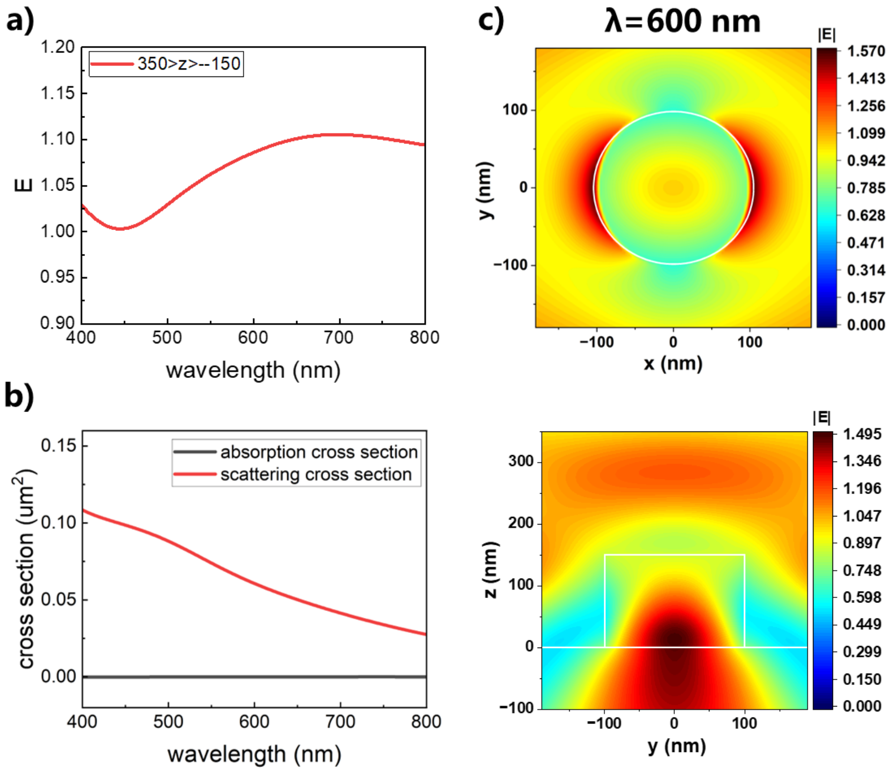


Figure S 4: (a) Electric field intensity integrated over the simulated volume for a single $TiO_2$ nanoparticle on the YAG:Ce substrate at $\theta_{\rm in} = 0°$. (b) Simulated forward scattering and absorption cross section at $\theta_{\rm in} = 0°$. (c) Electric field intensity map at $\lambda = 600$ nm. The upper panel shows the cross-section of $x - y$ plane at z = 20 nm, while the lower panel presents the $y - z$ plane through the center of the particle. The solid white lines indicate the geometric cross-section of the nanoparticle and the upper surface of substrate, while the area $z < 0$ nm stands for the substrate.

## 5 Simulated field distribution for the nanoparticle array

The electric field distribution of the nanoparticle array was further analyzed. At $\theta_{\text{in}} = 0°$, a dipole Mie resonance, and a standing wave-like diffraction coupling each Mie resonance, are observed at RA conditions ($\lambda = 515$ and $661$ nm). In the electric field distributions on the $y - z$ plane (lower panels of Figs. S5a and b), the coupling is mostly confined to the medium that satisfies the corresponding RA condition, i.e., the standing waves are evident in the PDMS and YAG:Ce side at $\lambda = 515$ and $661$ nm, respectively.

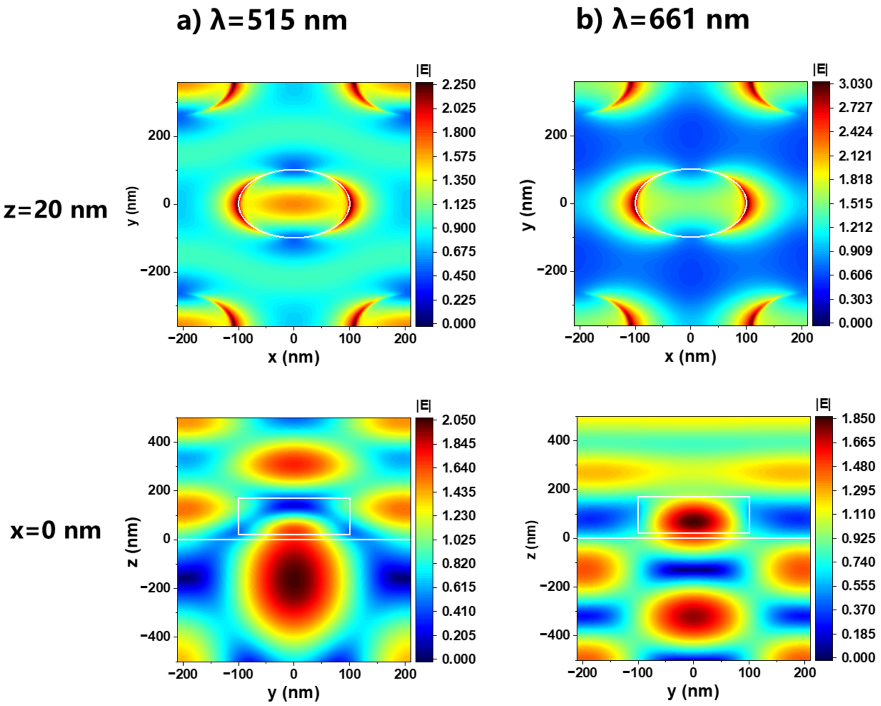


Figure S 5: Electric field intensity distribution at (a) $\lambda = 515$ nm and (b) $\lambda = 661$ nm for the nanoparticle array (hexagonal lattice, period = 420 nm). The upper panel shows the $x - y$ cross-section at $z = 20$ nm, where the bottom surfaces of the nanoparticles are located. The lower panel shows the cross-section of the $y - z$ plane. A 20 nm PDMS layer is placed between the nanoparticles and the YAG:Ce substrate.

# 6 Comparison of DBR spectra between experiment and simulation

Figure S6 shows the optical transmittance of the YAG:Ce plates with 0–, 3–, and 6–layer DBR. For the plate without DBR, the transmittance dips appears at $\lambda = 330$ and 460 nm, which are $f - d$ electronic transitions of $Ce^{3+}$. For the plate with 3-layer DBR, additional dips appear at $\lambda = 400$ and 560 nm, which are reflectance bands by the DBR. The dip at $\lambda =$ 560 nm is broad and covers the emission band of YAG:Ce. It is also noted the transmittance at around 460 nm, which is the excitation wavelength, does not change much, meaning the DBR less affects the excitation process. For the 6-layer DBR, the reflectance band at $\lambda =$ 560 nm further develops.

This reflectance was reproduced by numerical simulation using FDTD in Figs. S6 b and c. The experimental reflectance bands, shown in Fig. S6a and Figs. S3b and c, are reproduced.

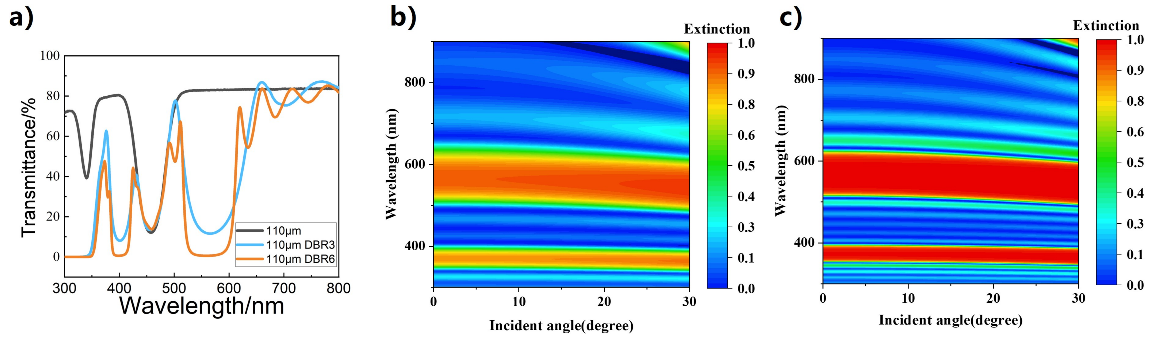


Figure S 6: (a) Uv-vis diagram of 110$\mu$m-thick substrate with 0-,3-,and 6-layer DBR (b-c) Simulation extinction spectra for (b)3-layer and (c) 6-layer DBR

# 7 Analysis of thickness dependence of PL directionality

Let us consider the situation where the phosphor plate is excited at a point on the bottom (x = z = 0). The PL radiates spherically in all directions and is detected with a finite size spot at the plate surface (see the Figure below). For the flat plate, the PL within the light cone is detected and those outside the cone are trapped and cannot be detected. In contrast, for the plate with antenna, the PL outside the cone turns to be directional component of the output. Thus, thinner plates containing more trapped PLs exhibit a narrower angular profile.

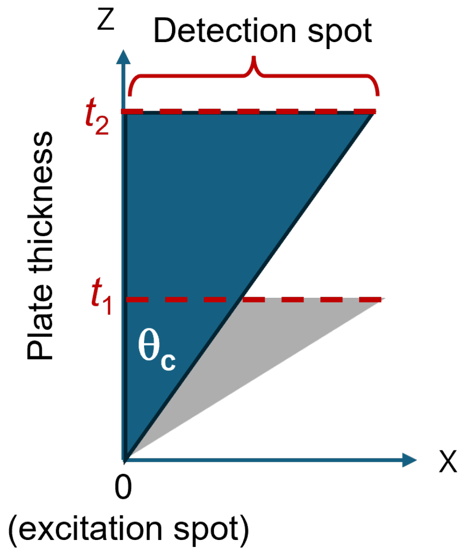


Figure S 7: Schematic of the relation between plate thickness, transmitted and trapped PLs from a point source. Thinner plates ($t_1$ in this figure) contain more trapped PLs inside the detection spot, which turn into a directional component for antenna samples.

# 8 Simulated PL enhancement of the square pattern nanoantenna sticker

We simulated the PL enhancement for a square patterned nanoantenna sticker with a period of 390 nm. Figure S8a shows that the PL enhancement is extended to larger emission angles than that of the hexagonal pattern, which is consistent with the experimental observations that the directionality is less evident for the square lattice (Fig. 3). Also the magnitude of simulated PL enhancement, up to 1.4, is smaller than that for the hexagonal lattice, up to 1.5 (Fig. 5). This qualitatively aligns with the experimental observations. Figure S8b compares the PL enhancements at $\theta_{em} = 0°$. The simulated spectral profile follows the experimental profile. The discrepancies at $\lambda \leq 500$ nm and $\lambda \geq 700$ nm come partly from the properties of phosphor (YAG:Ce) that mainly emits between 500 nm $\leq \lambda \leq$ 700 nm, and partly from deviations in shape of the fabricated nanoparticles from the designed geometry.

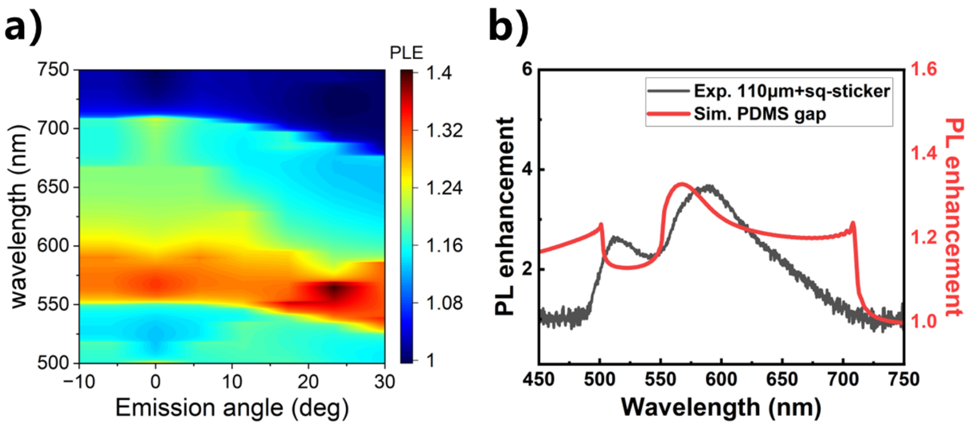


Figure S 8: (a) Simulated PL enhancement spectrum of square pattern sticker on the YAG:Ce substrate. (b) Comparison of the experimental PL enhancement using a 110 $\mu$m-thick substrate with the square pattern sticker (gray line) and the simulated result (red line) at $\theta_{em}$=0°.

# 9 Influence of PDMS layer on PL Enhancement

The effect of the PDMS layer on PL enhancement was investigated through numerical simulations. In this model, the $TiO_2$ nanoparticle array was constructed directly on the YAG:Ce substrate, and the superstrate material was varied to represent either the nanoantenna sticker or the directly fabricated nanoantenna on the substrate. The PDMS superstrate exhibited a sharper resonance peak around 661 nm and a stronger integrated PL intensity within the wavelength range of $515 - 661$ nm at $\theta_{em} = 0°$, which demonstrates the effectiveness of this sticker-based nanoantenna system.

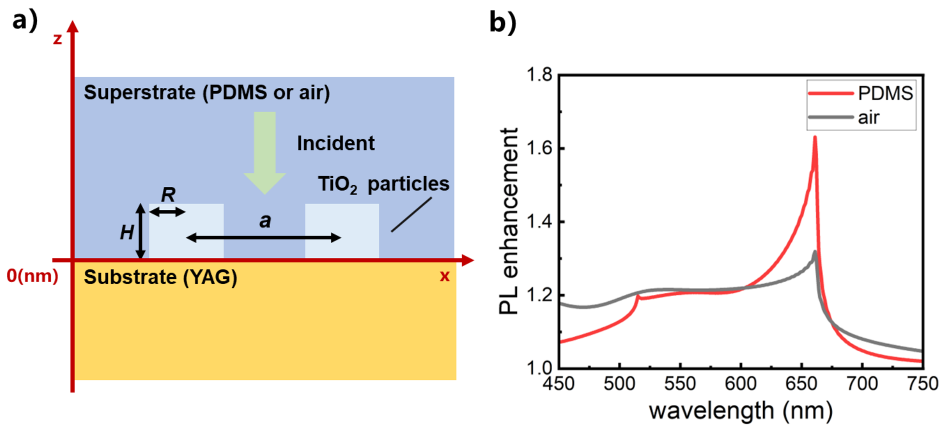


Figure S 9: (a) Schematic of the simulation structure of the $TiO_2$ nanoantenna sticker directly fabricated on YAG:Ce substrate. (b) Comparison of the PL enhancement of the YAG:Ce substrate with nanoantennas when covered by different superstrates (PDMS or air) at 0°.
